# Screening of Tanzanian medicinal plants for anti-Candida activity

**DOI:** 10.1186/1472-6882-6-11

**Published:** 2006-03-30

**Authors:** Deborah KB Runyoro, Mecky IN Matee, Olipa D Ngassapa, Cosam C Joseph, Zakaria H Mbwambo

**Affiliations:** 1 Department of Pharmacognosy, School of Pharmacy, Muhimbili University College of Health Sciences P.O Box 65013, Dar Es Salaam, Tanzania; 2 Department of Microbiology and Immunology, School of Medicine, Muhimbili University College of Health Sciences, P. O Box 65001, Dar Es Salaam, Tanzania; 3 Department of Chemistry, University of Dar Es Salaam, P.O Box 35065, Dar Es Salaam, Tanzania; 4 Institute of Traditional Medicine, Muhimbili University College of Health Sciences, P. O Box 65001, Dar Es Salaam, Tanzania

## Abstract

**Background:**

*Candida albicans *has become resistant to the already limited, toxic and expensive anti-*Candida *agents available in the market. These factors necessitate the search for new anti-fungal agents.

**Methods:**

Sixty-three plant extracts, from 56 Tanzanian plant species obtained through the literature and interviews with traditional healers, were evaluated for anti-*Candida *activity. Aqueous methanolic extracts were screened for anti-*Candida *activity by bioautography agar overlay method, using a standard strain of *Candida albicans *(ATCC 90028).

**Results:**

Twenty- seven (48%) out of the 56 plants were found to be active. Extracts of the root barks of *Albizia anthelmintica *and *Balanites aegyptiaca*, and roots of *Plectranthus barbatus *showed strong activity.

**Conclusion:**

The extracts that showed strong anti-*Candida *activity are worth of further investigation in order to isolate and identify the active compounds.

## Background

Numerous studies have shown an association between increased prevalence of HIV infection and the occurrence of opportunistic fungal infections [1,2]. Among the different HIV-associated fungal infections, oral mucosal lesions caused by *Candida *species are by far the most frequent manifestation [3]. Up to 90% of HIV-infected individuals suffer at least one episode during the course of their disease [3], and the incidence and severity of the episodes increase with decreasing immunity, especially when CD4+ cell counts fall to levels below 200 cells/mm^3 ^[4]. *Candida albicans*is the most causative agent, accounting for more than 90% of cases [5]. However, other *Candida *species such as *C. glabrata, C. parapsilosis, C. tropicalis*, and *C. krusei*may also cause symptomatic oral candidiasis in HIV-positive individuals [6]. Other fungal infections seen in HIV-infected individuals include, cryptococcosis due to *Cryptococcus neoformans *and aspergillosis due to *Aspergillus flavus*, *A. fumigatus *and *A. niger *[7,8].

In sub Saharan Africa, where 70% of the world cases of HIV/AIDS are found [9], oral candidiasis, including oropharyngeal and oesophageal candidiasis, are very common, causing significant morbidity among patients. Oral candidiasis is usually treated by topical antifungal agents, which include nystatin, miconazole, fluconazole, itraconazole and amphotericin B. However, the management of *Candida *infections faces a number of problems including; limited number of effective antifungal agents [10], toxicity of the available antifungal agents [10-12], resistance of *Candida *to commonly used antifungals [13-16], relapse of *Candida *infections [17], and the high cost of antifungal agents [10-12]. When relapses occur, the infections tend to be increasingly refractory to treatment. The difficulties associated with the management of *Candida *infections necessitate the discovery of new antifungal agents, in order to widen the spectrum of activity against *Candida *and combat strains expressing resistance to the available antifungal agents.

Plant-derived natural products may offer potential lead to new compounds, which could act on these fungi [18]. This paper reports on the screening of 63 aqueous methanol plant extracts for activity against *Candida albicans*. The extracts were obtained from 56 plant species, belonging to 29 plant families, collected from four regions of Tanzania including Coast, Dar es Salaam, Morogoro and Tanga. Screening was achieved by using the bioautography agar overlay method, which is very convenient, simple and efficient [19]. The method can also be employed in the target-directed isolation of the active constituents.

## Methods

### Plant collection

**Figure 1:**
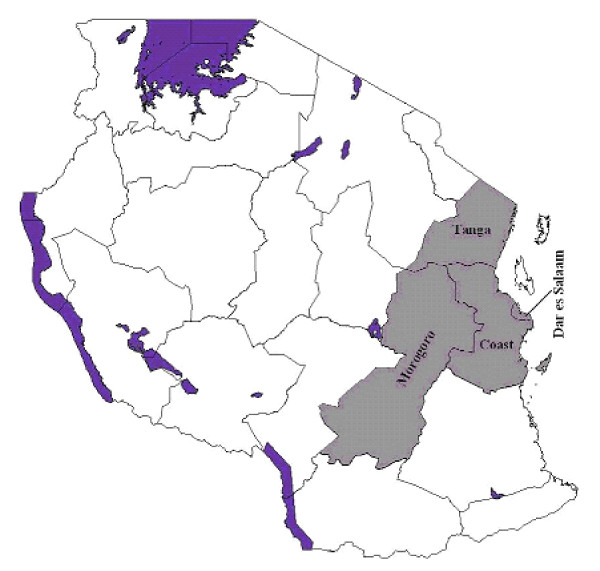
Map of Tanzania showing the regions where plants were collected.

Plants were collected from four regions, which are in the eastern part of Tanzania, namely, Coast, Dar es Salaam, Morogoro and Tanga (Figure 1). The plants included those reported in the literature [20,21] to be active against *Candida albicans*, but which have not been investigated further to identify the active compounds. The second category comprised of plants obtained through interviews with traditional healers.

### Interviews with traditional healers

Traditional healers in the four regions of Tanzania (Figure 1) were interviewed on plants they used to treat *Candida *infections. Symptoms of the various forms of *Candida *infections associated with HIV/AIDS were described to the traditional healers so as to enable them give the appropriate plants they used in the management of these conditions. The symptoms which have been described in the literature [22] include; oral thrush, mouth ulcers and lesions of epithelial cells of the lips, erythematous lesions on the dorsum of the tongue and angular cheilitis. Those for *Candida *oesophagitis were painful swallowing, a feeling of obstruction on swallowing, substernal chest pain and discrete ulceration of the oesophagus [23]. The symptom for vaginal candidiasis was a cuddle milk discharge [24].

Prior to the interview each traditional healer was asked to sign an agreement/consent form if she/he agreed with the terms given in the forms. The form, in short, explained the importance of the information they were providing and the type of research that was to be done on the plants they provided. They were also informed that the results and any profitable outcome would be communicated to them. This was done in order to safeguard the interests of both the parties.

### Identification of the plants

Preliminary identification of the plants was done in the field by a botanist. Herbarium specimens were prepared and photographs were taken to aid in the confirmation of the identity of the plants. Voucher specimens were deposited in the Herbarium of the Botany Department, University of Dar es Salaam, Tanzania, where identity of the plants was confirmed by comparison with available voucher specimens.

### Processing of the plant materials

The plant materials were sorted, washed and chopped into smaller pieces, where necessary, before drying. The materials were dried outdoors; however, leaves were dried in the sun for one day, then in the shade. The dried plant materials were ground to various degrees of fineness depending on their botanical structures. This was done using a Fitz mill Type 6 (Manesty machines Ltd. Liverpool, England).

### Screening of the extracts for anti-Candida activity

#### Preparation of extracts and standards

Ground plant materials (100 to 200 g) were macerated with 80% methanol at room temperature (28°C). In each case the amount of solvent used was just enough to cover the plant materials. Maceration was carried out for two days and the procedure was repeated three times. Extracts were decanted then filtered using filter funnels fitted with Whatman No 1 filter papers. The extracts were pooled and concentrated using a Büchi rotary evaporator (Büchi Labortechnik, Flawil, Switzerland) set at 40–50°C, followed by freeze-drying using the Edwards freeze drier (Edwards High Vacuum International Crawley, Sussex, England). For each extract 1 g was weighed and dissolved in 5 ml of methanol to produce a solution with a concentration of 200 μg/μl.

Amphotericin B (Calbiochem, USA and Canada) was used as the standard anti-fungal agent.

#### Inoculum

Sabouraud dextrose agar (SDA; Biotec Laboratories Ltd. UK) was used to prepare the culture medium according to the manufacturer's directions. *Candida albicans *ATCC 90028 (provided by Prof. Paul Verweij, Department of Medical Microbiology, University Medical Center, St. Radboud Hospital, Nijmegen, The Nertherlands) was aseptically inoculated on petridishes containing autoclaved, cooled and settled medium. The petridishes were incubated at 31°C for 48 h to give white round colonies against a yellowish background. These were aseptically sub-cultured on SDA slants.

*Candida albicans *colonies from SDA slants were suspended in sterilised 0.9% sodium chloride solution (normal saline), which was compared with a 5 McFarland solution. The microbial suspension (1 ml) in normal saline was added to 74 ml of sterile medium, kept at 45°C, to give concentration of 2 × 10^7 ^cells/ml.

### Bioautography agar overlay

#### Preamble

The bioautography agar overlay method is an improved version of a disc diffusion method. Unlike the ordinary diffusion method in which the drug to be evaluated is adsorbed on a paper disc and placed onto the inoculated petridish, in the bioautography agar overlay method the drug to be evaluated is adsorbed onto the Thin Layer Chromatography (TLC) plate and the inoculum is laid onto the plate as a very thin layer of about 1 mm in thickness. In both cases the drug diffuses from the adsorbent into the inoculum where it exerts its effects. The bioautography agar overlay method is advantageous in that, firstly it uses very little amount of sample when compared to the normal disc diffusion method and hence it can be used for bioassay- guided isolation of compounds, and secondly, since the crude extract is resolved into its different components it simplifies the process of identification and isolation of the active compounds [19].

#### Bioautography agar overlay process

Five microliters (5 μl) of the solution of each extract was applied on a glass backed Silica gel G_60 _F_254 _TLC plates (20 × 20 cm, 250 μm thickness, Merck, Darmstadt, Germany) and reference plate was similarly prepared. Both plates were developed to a distance of about 10 cm in the same tank using the pre-determined mobile phases. The mobile phase was removed from the plate by drying with a stream of cool air from a heating gun. The reference plate was observed in UV light to see if the separated spots were UV active after which it was sprayed with vanillin sulphuric acid followed by heating using a heating gun.

The positive control, amphotericin B (0.002 μg/μl) equivalent to 0.01 μg per spot was applied on the test plate in triplicate followed by removal of solvent using a stream of cold air. About 22 ml of the freshly prepared inoculum described above was uniformly spread using a sterile Pasteur pipette. After solidification the plates were placed in a polythene container lined at the bottom, with moist cotton wool. The plates were incubated at 31°C for 18 to 24 h, after which they were removed and sprayed with an aqueous solution (2.5 mg/ml) of thiazolyl blue (3-(4,5 dimethylthiazolyl-2)-2, 5-diphenyl tetrazolium bromide) (MTT) (BDH, Poole England). They were incubated for a further 4 h, after which the inhibition zones appeared colourless against a purple background. Spots showing any inhibition were noted and their hRf values and inhibition zones measured. The test was performed in duplicate.

## Results and discussion

**Figure 2:**
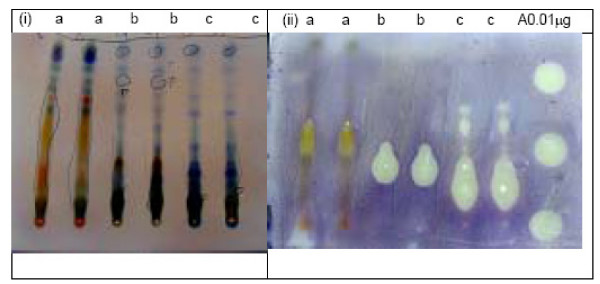
Chromatograms (i) and bioautograms (ii) for *Holarrhena febrifuga *leaves (a), *Balanites aegyptiaca *root bark (b) and *Albizia anthelmintica *root bark (c). Mobile phase: Chloroform:Methanol:Ethylacetate:water 28:30:35:5; Amphotericin B (A)

**Figure 3:**
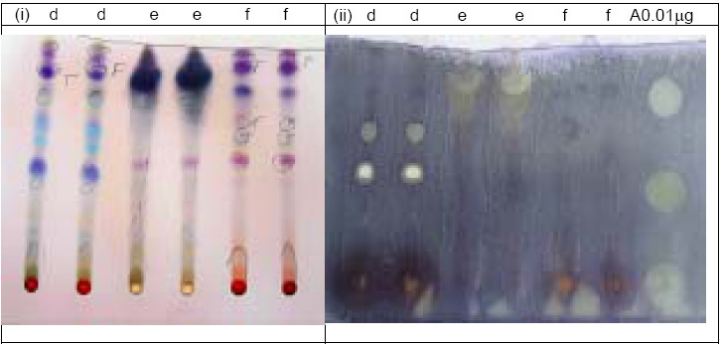
Chromatograms (i) and bioautograms (ii) for *Combretum zeyheri *leaves (d), *Plectranthus barbatus *roots (e) and *Vitex fischeri *root bark (f) mobile phase: Chloroform: Methanol 4:1 3; Amphotericin B (A).

**Figure 4:**
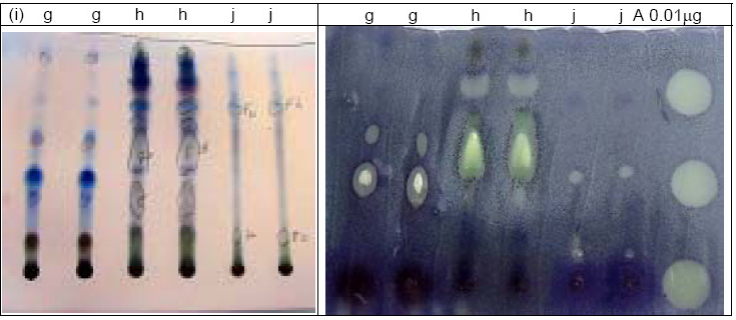
Chromatograms (i) and bioautograms (ii) for *Margaritaria discoidea *roots (g), *Cajanus cajan *leaves (h) and *Zanha africana *roots (j) mobile phase: Chloroform: Methanol 4:1 3; Amphotericin B (A).

The plants studied gave aqueous methanolic extracts with yields ranging from 2 to 26.5% (Table 1). Twenty-eight out of the sixty-three extracts, belonging to 27 plant species and constituting 48% of all the plants collected, could be resolved into TLC spots exhibiting activity against *C. albicans *(Table 1). The number of active resolved spots for each extract ranged from 1 to 4. Table 1 shows the TLC bioautography results for the various extracts developed using various solvent systems and Figures 2, 3, 4 show the bioautograms of some of the most active extracts.

**Table 1:** TLC Bioautography results of various extracts.

**S/N**	**Botanical Name**	**Family**	**Source**	**Plant part**	**% Yield of the Extract**	**Mobile Phase**	**Average hRrf value**	**Average inhibition zones in mm**
1	*Abrus precatorius *L.	Fabaceae	T	L	18.3	3	-	-
2	*Acacia nilotica *(L.) Del.	Fabaceae	T	Rb	16.5	3	63	< 4
							75	< 4
							85	< 4
3	*Acacia zanzibarica *(S. Moore) Taub.	Fabaceae	T	Rb	12.5	2	-	-
4	*Agathisanthemum bojeri *Klotzsch	Rubiaceae	T	Ap	6.5	3	-	-
5	*Agathisanthemum bojeri *Klotzsch	Rubiaceae	T	R	16.0	4	80	< 4
							100	10
6	*Albizia anthelmintica *Brongn	Fabaceae	T	Rb	9.0	5	25	15
							40	4
							50	5
							60	6
7	*Allophyllus africanus *P. Beauv.	Sapindaceae	T	Ap	3.0	3	-	-
8	*Asparagus africanus *Lam.	Liliaceae	Lit*	Ap	16.4	2	0	< 4
							50	< 4
							75	< 4
9	*Balanites aegyptiaca *(L.) Delile	Zygophyllaceae	T	Rb	22.0	5	30	13
10	*Bonamia mossambicensis *Klotzsch	Convolvulaceae	Lit*	R	6.0	3	-	-
11	*Boscia salicifolia *Oliv.	Capparaceae	Lit*	L	14.5	4	-	-
12	*Bridelia cathartica *Bertol.	Euphorbiaceae	Lit*	Sb	10.0	2	-	-
13	*Cajanus cajan *(L.) Millsp	Fabaceae	T	L	8.0	3	37	4
							53	10
							78	10
14	*Carica papaya *L.	Caricaceae	T	R	6.0	3	-	-
15	*Cassia abbreviata *Oliv	Fabaceae	T	Rb	11.5	1	-	-
16	*Cassia auriculata *L.	Fabaceae	Lit*	Sb	10.0	4	60	10
							85	8
17	*Catunaregum nilotica *(Stapf) Tier.	Rubiaceae	T	Rb	6.0	3	0	< 4
							40	< 4
							55	< 4
							80	< 4
18	*Ceiba pentandra *Gaertn	Bombacaceae	Lit*	L	5.5	3	-	-
19	*Chassalia umbraticola *Vatke	Rubiaceae	T	Rb	6.0	3	0	5
							*80*	6
20	*Clutia abyssinica *Jaub. & Spach	Euphorbiaceae	T	L	9.5	1	-	-
21	*Combretum molle *R. Br.	Combretaceae	T	R	13.5	4	33	7
							48	4
22	*Combretum zeyheri *Sond	Combretaceae	Lit*	L	3.5	3	46	9
							61	6.5
23	*Crabbea velutina *S. Moore	Acanthaceae	T	W	8.0	3	-	-
24	*Deinbollia borbonica *Scheff	Sapindaceae	Lit*	R	5.0	3	-	-
25	*Dichrostachys cinerea *(L.) Wight & Arn	Fabaceae	T	L	12.0	1	-	-
26	*Dictyophleba lucida *(K. Schum.) Pierre	Apocynaceae	Li	L	13.0	2	10	5
							40	5
							85	<4
27	*Ehretia amoena *Klotzsch	Boraginaceae	T	Sb	6.5	3	-	-
28	*Elaeodendron buchananii *(Loes) Loes	Celastraceae	T	Rb	13.5	3	-	-
29	*Eriosema psoraleoides *(Lam.) G. Don	Fabaceae	Li	Sb	4.5	3	-	-
30	*Flueggea virosa *(Roxb. ex Wild.) Voigt.	Euphorbiaceae	Lit**	S	2.5	3	-	-
31	*Harrisonia abyssinica *Oliv.	Simaroubaceae	Lit*&T	Rb	12.5	1	90	10
32	*Harrisonia abyssinica *Oliv.	Simaroubaceae	Lit*	Ap	10.5	1	-	-
33	*Hibiscus micranthus *L. f.	Malvaceae	Lit*	Ap	3.5	3	-	-
34	*Holarrhena febrifuga *Klotzsch	Apocynaceae	Lit*	L	13.0	5	45	10
35	*Lannea stuhlmannii *Engl.	Anacardiaceae	T	R	9.5	3	-	-
36	*Margaritaria discoidea *(Baill) G.L. Webster	Euphorbiaceae	T	R	15.0	3	50	5
							60	4
37	*Ocimum suave *Willd	Lamiaceae	T	R	5.5	3	-	-
38	*Ozoroa insignis *Delile (from Morogoro)	Anacardiaceae	T	Rb	11.5	1	-	-
39	*Ozoroa insignis *Delile (from Tanga)	Anacardiaceae	T	Rb	9.0	3	-	-
40	*Phyllanthus reticulatus *Poir	Euphorbiaceae	Lit*	Ap	11.5	1	-	-
41	*Physalis peruviana *L.	Solanaceae	T	L	13.6	1	0	7
							45	6
							80	< 4
42	*Plectranthus barbatus *Andrews	Lamiaceae	T	R	5.5	3	72	12.5
43	*Plectranthus barbatus *Andrews	Lamiaceae	T	L	2.0	1	85	7
44	*Pseudolachnostylis maprouneaefolia *Pax	Euphorbiaceae	Lit*	S	12.0	4	-	-
45	*Pseudolachnostylis maprouneaefolia *Pax	Euphorbiaceae	Lit*	R	12.0	1	-	-
46	Pseudovigna argentea (Willd) Verde	Fabaceae	T	L	4.0	3	Sf	<4
47	*Salvadora persica *L.	Salvadoraceae	T	Rb	8.5	3	80	10
48	*Sclerocarya birrea *(A. Rich) Hochst	Anacardiaceae	T	Rb	9.0	3	-	-
49	*Securidaca longepedunculata *Fres.	Polygalaceae	T	Rb	26.5	3	72	< 4
							85	< 4
50	*Sida serratifolia *L.	Malvaceae	Lit*	Ap	4.0	1	85	10
51	*Suregada zanzibariensis *Baill.	Euphorbiaceae	T	L	8.0	1	0	10
							80	5
							100	< 4
52	*Synoptolepis kirkii *Oliv	Thymelaeaceae	T	R	5.7	3	-	-
53	*Tetracera boiviniana *L.	Dilleniaceae	T	L	4.0	3	-	-
54	*Uvaria acuminata *Oliv.	Annonaceae	T	R	7.0	3	70	7
55	*Vitex fischeri *Gürke	Verbenaceae	Lit*	Rb	6.0	3	52	< 4
							65	< 4
56	*Xeroderris stuhlmannii *(Taub) Mendonca	Fabaceae	Lit*	Sb	6.5	3	-	-
57	*Ximenia americana *L. (from Morogoro)	Olacaceae	T	Rb	18.5	4	-	-
58	*Ximenia americana *L. (from Tanga)	Olacaceae	T	Rb	8.5	4	-	-
59	*Zanha africana *(Radlk.) Exell.	Sapindaceae	T	R	6.5	3	11	4
							41	5
60	Zanthoxylum chalybeum Engl.	Rutaceae	Lit*	Rb	9.0	4	30	< 4
							85	< 4
61	*Ziziphus abyssinica *Hochst. ex A. Rich.	Rhamnaceae	Lit*	L	11.5	6	37	9
62	*Ziziphus abyssinica *Hochst. ex A. Rich.	Rhamnaceae	Lit*	Sb	9.0	3	-	-
63	*Ziziphus mucronata *Willd	Rhamnaceae	T	R	8.0	3	75	< 4

Key:

Ap: aerial parts; L: leaves; Lit: literature; R: roots; Rb: root bark; S: stem; Sb: stem bark; Sf: several faint spots, T: traditional healer; W: whole plant, -: no inhibition, *Sawhney et al. (20), **Khan et al. (21),

Mobile phases

1: Chloroform: Methanol 9:1

2: Chloroform:Methanol 5:1

3: Chloroform:Methanol 4:1

4: Chloroform:Methanol:Ethylacetate 4:1:1

5: Chloroform:Methanol:Ethylacetate:water 28:30:35:5

6: Dichloromethane:Methanol:Water 30:10:1

Bioautography agar overlay method is considered as one of the most efficient methods for the detection of antimicrobial compounds [19]. It involves the transfer of the active compounds from the stationary phase into the agar layer through a diffusion process. From Table 1 it can be seen that a total of 27 plants, screened by using this method, exhibited anti-*Candida *activity. However, only 8 out of the 18 plants previously reported to be active by Sawhney et al. [20], were found to be active in this bioassay. *Ziziphus abyssinica *and *Asparagus africanus *which were collected as substitutes for *Ziziphus pubescens *and *Asparagus falcatus *respectively (reported active by Sawhney et al. [20] were also found to be active. This proves the fact that allied species may contain similar or related active compounds [25]. *Eriosema psoraleoides*, a plant, reported by Khan et al. [21] to be active was found to be inactive in this bioassay.

No obvious reasons can be given as to why some of the plants reported previously to be active were found inactive in this study. The plant parts used were more or less similar, with very few peculiar exceptions, such as *Xerroderris stuhlmanii*, which Sawhney et al. [20] reported that the whole plant was active. However this plant is a tree, and, in this study only the stem barks was evaluated. The bioassay method employed in this study is quite different from the one used by Sawhney et al. [20]. In the present study the bioautography method was used on partially resolved extracts, whereas Sawhney et al. [20] used the agar dilution method for unresolved crude extracts. Also it was not possible to compare concentrations because in the bioautography method absolute amounts of extracts were chromatographed while in the agar dilution method concentrations of the extracts were used. Such differences could have contributed to the observed differences in the bioassay results.

Eighteen (49%) out of 37 plants, obtained through interviewing traditional healers, were found active in this bioassay. Ten of these have also been reported to be active in previous studies; these include; *Ziziphus mucronata *[26], *Cajanus cajan *[27], *Acacia nilotica *[28], *Harrisonia abyssinica *and *Zanthoxylum chalybeum *[20]. Others were *Securidaca longepedunculata*, [29,30]*Salvadora persica *[31], *Balanites aegyptiaca *[32], *Zanha africana *[33], *Combretum molle *[21]. *Harrisonia abyssinica *and *Z. chalybeum *were selected through the literature and were also mentioned by the interviewed traditional healers. *Physalis peruviana*, was reported previously as inactive [34], but was found to be active in this study. The remaining eight active plants, including *Margaritaria discoidea, Suregada zanzibariensis, Plectranthus barbatus, Albizia anthelmintica, Securidaca longepedunculata, Catunaregum nilotica, Pseudovigna argentea, Agathisanthemum bojeri *and *Chassalia umbraticola*, were screened for anti- *Candida *activity for the first time. Other plants obtained from the traditional healers, which were reported active previously were found to be inactive in this study and they include *Ozoroa insignis *[35], *Carica papaya *[26] and *Dichrostachys cinerea *[28]. The discrepancy in the results could be due to a number of factors, including differences in the plant parts used, extraction solvents, time of collection of plant materials, geographical location of the plants and bioassay methods.

Eleven plants were resolved into several spots showing large inhibition zones (≥ 10 mm). The plants included *Agathisanthemum bojeri, Albizia anthelmintica*, *Balanites aegyptiaca, Cajanus cajan, Cassia auriculata, Harrisonia abyssinica*,*Holarrhena febrifuga, Plectranthus barbatus*, *Salvadora persica, Sida serratifolia *and *Suregada zanzibariensis*. Eight among these were obtained through interviewing traditional healers including *A. anthelmintica, B. aegyptiaca *and *P. barbatus *the most active of the screened plants. This observation shows that ethnomedical information is important in drug discovery.

*Albizia anthelmintica *and *Balanites aegyptiaca *have been reported to contain several triterpene saponins and steroidal saponins, respectively [36-38]. Also the extracts of these plants formed strong foam, with water indicating that they contain saponins. The presence of saponins in these plants is also supported by the chromatogram obtained by visualizing with vanillin-sulphuric acid (Fig 2) in which the probable active compounds gave purple or blue colour with this reagent, a normal positive test for triterpenoidal and steroidal saponins respectively [39]. The roots of *B. aegyptiaca *have, also been reported to contain flavonoids [40]. The root bark of *A. anthelmintica *was reported to contain a histamine alkaloid and proteins [41]. A. *anthelmintica *was screened for anti-*Candida *activity for the first time and, although *B. aegyptiaca *was reported to be active in the previous study [32] no identification of compounds responsible for the activity was done.

*Plectranthus, barbatus *another promising plant has been reported to contain diterpenes [42] and an essential oil containing α-pinene, myrcene and caryophylene as the major constituents [43]. The plant has never been evaluated for anti-*Candida *activity before, and thus, it has been shown for the first time in this study to have anti-*Candida *activity.

## Conclusion

Almost 50% of the collected plants were found to be active against *Candida albicans *(ATCC 90028) with varied degrees of activity. About half (49%) of the plants, obtained by interviewing traditional healers, demonstrated activity against *C. albicans*. This study gives an indication of the efficacy of the plants obtained from the traditional healers. The results from this study form a basis for further studies on the active plants so as to isolate the compounds responsible for the observed anti-*Candida *activity.

## Abbreviations

1. ATCC: American Type Culture Collection

2. BDH: British Drug House

3. CD4: Cluster Designation 4

4. HIV/AIDS: Human Immunodeficiency Virus/Acquired Immune Deficiency Syndrome

5. ITM: Institute of Traditional Medicine

6. MTT: Methylthiazolyltetrazolium Chloride

7. MUCHS: Muhimbili University College of Health Sciences

8. SDA: Sabouraud dextrose agar

9. TLC: Thin layer Chromatography

10. UDSM: University of Dar es Salaam

## Authors' contributions

Deborah KB Runyoro : Conception and designing of the research, acquisition of data, drafting the manuscript and revising it critically for important intellectual content.

Mecky IN. Matee: Conception and designing of the research and revising the manuscript critically for important intellectual content and gave final approval for its publication.

Olipa D Ngassapa: Conception and designing of the research and revising it critically for important intellectual content and gave final approval for its publication.

Cosam C Joseph: Conception and designing of the research and revising it critically for important intellectual content.

Zakaria H Mbwambo: Acquisition of data and revising the manuscript critically for important intellectual content.

## Pre-publication history

The pre-publication history for this paper can be accessed here:


